# An integrated micromachined flexible ultrasonic-inductive sensor for pipe contaminant multiparameter detection

**DOI:** 10.1038/s41378-024-00734-0

**Published:** 2024-08-16

**Authors:** Zheng Yuan, Xiaoyu Wu, Zhikang Li, Jiawei Yuan, Yihe Zhao, Zixuan Li, Shaohui Qin, Qi Ma, Xuan Shi, Zilong Zhao, Jiazhu Li, Shiwang Zhang, Weixuan Jing, Xiaozhang Wang, Libo Zhao

**Affiliations:** 1 State Key Laboratory for Manufacturing Systems Engineering, International Joint Laboratory for Micro/Nano Manufacturing and Measurement Technologies, Xi’an Jiaotong University (Yantai) Research Institute for Intelligent Sensing Technology and System, Xi’an Jiaotong University, Xi’an, 710049 China; 2 School of Mechanical Engineering, Xi’an Jiaotong University, Xi’an, 710049 China; 3 School of Instrument Science and Technology, Xi’an Jiaotong University, Xi’an, 710049 China; 4 Shandong Laboratory of Yantai Advanced Materials and Green Manufacturing, Yantai, 265503 China

**Keywords:** Physics, Engineering

## Abstract

Pipe contaminant detection holds considerable importance within various industries, such as the aviation, maritime, medicine, and other pertinent fields. This capability is beneficial for forecasting equipment potential failures, ascertaining operational situations, timely maintenance, and lifespan prediction. However, the majority of existing methods operate offline, and the detectable parameters online are relatively singular. This constraint hampers real-time on-site detection and comprehensive assessments of equipment status. To address these challenges, this paper proposes a sensing method that integrates an ultrasonic unit and an electromagnetic inductive unit for the real-time detection of diverse contaminants and flow rates within a pipeline. The ultrasonic unit comprises a flexible transducer patch fabricated through micromachining technology, which can not only make installation easier but also focus the sound field. Moreover, the sensing unit incorporates three symmetrical solenoid coils. Through a comprehensive analysis of ultrasonic and induction signals, the proposed method can be used to effectively discriminate magnetic metal particles (e.g., iron), nonmagnetic metal particles (e.g., copper), nonmetallic particles (e.g., ceramics), and bubbles. This inclusive categorization encompasses nearly all types of contaminants that may be present in a pipeline. Furthermore, the fluid velocity can be determined through the ultrasonic Doppler frequency shift. The efficacy of the proposed detection principle has been validated by mathematical models and finite element simulations. Various contaminants with diverse velocities were systematically tested within a 14 mm diameter pipe. The experimental results demonstrate that the proposed sensor can effectively detect contaminants within the 0.5−3 mm range, accurately distinguish contaminant types, and measure flow velocity.

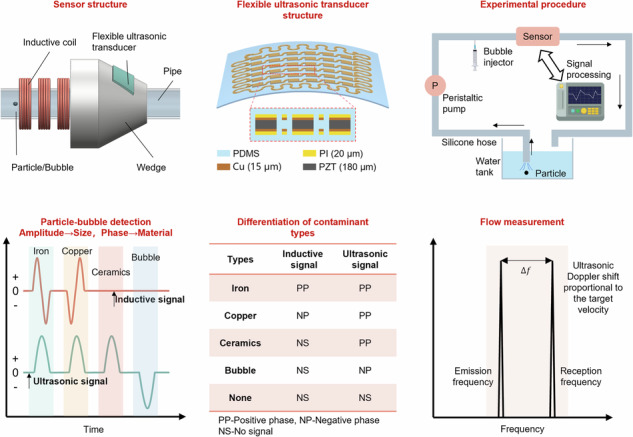

## Introduction

Oil is the blood of mechanical systems and is crucial for facilitating energy transfer, lubrication, sealing, and cooling processes^1^. Fluid contaminants from machining and assembly residues, equipment wear, and poor sealing include solid impurities, e.g., iron, copper, and ceramics, and nonsolid contaminants, e.g., air bubbles^2^. These contaminants can indicate the wear situation and fluid quality. During the operation of equipment, mechanical system wear and fluid deterioration are inevitable. Therefore, monitoring fluid contaminants is essential for early failure detection, maintenance facilitation, and lifespan prediction^3,4^.

Currently, offline fluid monitoring techniques, such as spectroscopy, are widely used to provide detailed and accurate information regarding the state of equipment^5,6^. Nevertheless, offline monitoring falls short of providing real-time equipment condition updates. As machinery complexity and reliability demands increase, online monitoring has become increasingly essential for health management^7^. The detection principles for online monitoring methods are mainly categorized into optical, electrical, acoustic, and magnetic methods^8^. Optical methods use visual imaging to capture real-time images of wear particles, but image quality can be affected by oil transparency, water droplets, air bubbles, etc^9–12^. Electrical methods are used to monitor oil properties by analyzing changes in electrical physical quantities, specifically the resistance and capacitance of sensing elements^13,14^. Acoustic methods employ ultrasonic echoes to detect particles and bubbles. However, their complexity, high cost, and inability to distinguish particles pose inherent challenges^15,16^. The inductive method can be utilized to determine the particle size and material by detecting perturbations in a magnetic field. Despite its high precision, it is exclusive to metal particle detection^17^.

Distinguishing the particle size and material within a pipeline is instrumental for assessing equipment wear and identifying its specific location^18,19^. The detection of bubbles and flow rates is essential for evaluating fluid quality and equipment performance^20,21^. H. Zhang et al.^22^ introduced a theoretical model for the inductance and resistance of a single-coil sensor, successfully distinguishing between iron and copper particles in a 900 µm diameter microfluidic channel. M. Qian et al.^23^ presented a triple-coil inductive sensor for detecting iron and copper particles in a 10 mm diameter pipe. This sensor features adjustable coil excitation to reduce heat while maintaining high sensitivity. H. Shi et al.^24,25^ proposed a capacitive-inductive microfluidic sensor installed on a 300 µm diameter microchannel to detect metal particles and bubbles simultaneously. To improve the throughput of microfluidic sensors, C. Wang et al.^26^ recommended a four-channel sensor, with each channel featuring its own inductive or capacitive element. Electrical methods can only detect metallic particles. Ultrasonic methods are generally used for nonmetallic particles. C. Xu et al.^27^ proposed ultrasonication to enhance particle detection by focusing the acoustic field within the detection region. L. Du et al.^28^ presented a combined electromagnetic-ultrasonic sensing method for detecting air bubbles and particles in pipelines. However, these methods commonly utilize microfluidic structures, which are not conducive to monitoring the original pipelines directly.

Most of the existing sensing methods are limited to monitoring a single parameter or commonly rely on microfluidic structures, which may be impractical in industrial settings. Additionally, the flow rate indicator should be considered. The flow rate is related to the equipment operating status and affects the particle detection error. To address the above issues, this paper proposes an integrated inductive-micromachined flexible ultrasonic sensor that can realize multiparameter online oil monitoring. This method can distinguish between ferromagnetic metal particles, nonferromagnetic metal particles, nonmetallic particles, and air bubbles while measuring the flow rate. Micromachining can significantly reduce the transducer size, whereas flexible substrates enable more accessible installation, enhancing focused acoustic fields for improved detection capabilities.

## Results and discussion

### Detection principle

**Fig. 1:**
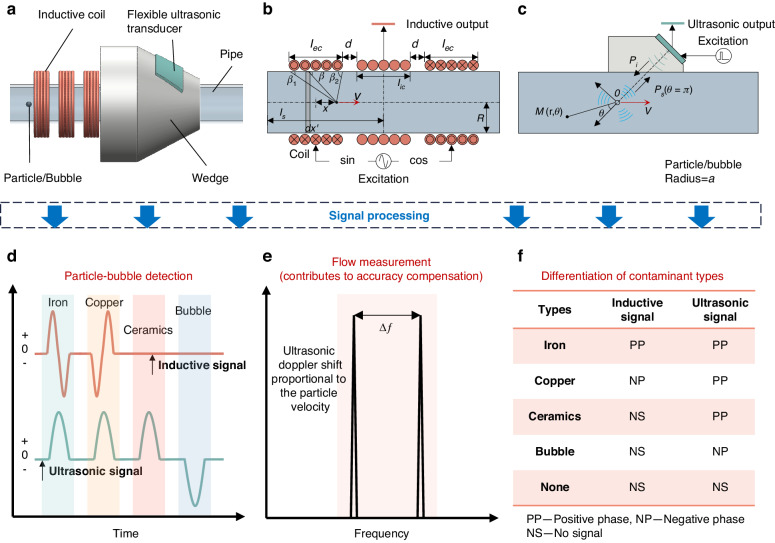
Working principle of the integrated induction-micromachined flexible ultrasound sensor. **a** Sensor structure, **b** electromagnetic inductive detection principle, **c** ultrasonic detection principle, **d** inductive and ultrasonic signals of different types of contaminants, **e** ultrasonic Doppler flow velocity measurement for accuracy compensation, and **f** rules for distinguishing different contamination types

Figure 1a illustrates the monitoring principle. The pipe is equipped with electromagnetic inductive and flexible ultrasonic units. The inductive unit comprises three symmetrically distributed coils, with the two coils on either side employing inverted AC excitation. Simultaneously, the middle coil monitors the metal particles passing through the pipe by electromagnetic induction effects. Additionally, a flexible ultrasound patch is affixed to a wedge. The ultrasonic patch adopts a 6*6 array design, and the snake electrode is used to connect the array elements, which can enhance the patch’s deformation ability. The wedge is used to direct the ultrasonic signal into the fluid at a set angle, which facilitates better capture of contaminants against reflected sound waves.

The induction and ultrasonic signals of different contaminants can be obtained through signal processing, as illustrated in Fig. 1d. The inductive unit can effectively distinguish between magnetic and nonmagnetic metal particles (e.g., iron and copper) but cannot detect nonmetallic solid contaminants (e.g., ceramics) and air bubbles. On the other hand, the ultrasonic unit demonstrates sensitivity to all solid particles and excels in detecting air bubbles, yet it cannot differentiate between various particle types. Therefore, combining time-domain signals from both sensing units enables discrimination among magnetic and nonmagnetic metals, nonmetallic solid particles, and air bubbles. The contaminant-type determination rules are shown in Fig. 1f. This approach effectively identifies nearly all types of contaminants within a pipeline. Additionally, particle velocity can be determined by the Doppler shift of an ultrasound signal. The particle velocity can directly reflect the pipeline’s flow rate and effectively compensate for inaccuracies in particle inductive detection.

### Theoretical Model

In Fig. 1(b), the axial component of the magnetic field at any point $x$ generated from a coil can be conceptualized as the superposition of magnetic fields generated by each turn of the coil current. The magnetic field^29^ produced by a solenoid coil of length $l$ at this specific point can be represented as

1 $$\begin{array}{c}B=\int {dB}=\frac{{\mu }_{0}{IN}}{2}\left(\frac{\frac{l}{2}+x}{\sqrt{{R}^{2}+{\left(\frac{l}{2}+x\right)}^{2}}}-\frac{\frac{l}{2}-x}{\sqrt{{R}^{2}+{\left(\frac{l}{2}-x\right)}^{2}}}\right)\end{array}$$

where ${\mu }_{0}$ is the vacuum permeability, *I* is the current intensity, $N$ is the coil turns, and $R$ is the coil radius.

The magnetic field of the three-coil inductive unit is the superposition of the fields produced by the two excitation coils, and this relationship can be expressed as

2 $${B}_{0z}={B}_{0{z}_{{left}}}+{B}_{0{z}_{{right}}}=\frac{{\mu }_{0}{IN}}{2}\left\{\cos {\beta }_{1}\left({x}_{l}\right)-\cos {\beta }_{2}\left({x}_{l}\right)+\cos {\beta }_{1}\left({x}_{r}\right)-\cos {\beta }_{2}\left({x}_{r}\right)\right\}$$

where

3 $$\cos {\beta }_{1}=\frac{\frac{l}{2}+x}{\sqrt{{R}^{2}+{\left(\frac{l}{2}+x\right)}^{2}}},\cos {\beta }_{2}=\frac{\frac{l}{2}-x}{\sqrt{{R}^{2}+{\left(\frac{l}{2}-x\right)}^{2}}}$$

4 $$\begin{array}{ll}z={vt}-{l}_{s}-0.5h,{x}_{l}={vt}-\left({l}_{s}-d-\frac{1}{2}{l}_{{ec}}-\frac{1}{2}{l}_{{ic}}\right),\\{x}_{r}={vt}-\left({l}_{s}+d+\frac{1}{2}{l}_{{ec}}+\frac{1}{2}{l}_{{ic}}\right)\end{array}$$

In our previous work^30^, the scattered field magnetic vector potential ${A}_{s}$ of a spherical particle under the influence of an external magnetic field was obtained, which can be expressed as

5 $${A}_{s}=\frac{1}{2}\frac{\left(2{\mu }_{r}+1\right){qa}{J}_{\frac{3}{2}}\left({qa}\right)-{q}^{2}{a}^{2}{J}_{\frac{1}{2}}\left({qa}\right)}{\left({\mu }_{r}-1\right){qa}{J}_{\frac{3}{2}}\left({qa}\right)+{q}^{2}{a}^{2}{J}_{\frac{1}{2}}\left({qa}\right)}{B}_{0z}\sin \theta {a}^{3}{r}^{-2}$$

where ${q}^{2}=-j\omega {\mu }_{r}{\mu }_{0}\gamma$, $\omega$ is the coil excitation frequency, ${\mu }_{r}$ is the particle relative magnetic permeability, $\gamma$ is the particle conductivity, and ${J}_{n+1/2}\left(\cdot \right)$ is a Bessel function of the first kind.

The magnetic induction intensity $\varPhi$ can be obtained from the curl of the magnetic vector potential, and the magnetic flux is obtained by integrating the magnetic induction intensity.

6 $$\varPhi =\int \nabla \times {A}_{s}=\int \frac{\left(2{\mu }_{r}+1\right){qa}{J}_{\frac{3}{2}}\left({ka}\right)-{q}^{2}{a}^{2}{J}_{\frac{1}{2}}\left({ka}\right)}{2\left({\mu }_{r}-1\right){qa}{J}_{\frac{3}{2}}\left({ka}\right)+{q}^{2}{a}^{2}{J}_{\frac{1}{2}}\left({ka}\right)}\,\cdot\, {a}^{3}{r}^{-2}\left(3{\cos }^{2}\theta -1\right)$$

and the induced voltage is obtained from the change in magnetic flux:

7 $$u=N\frac{d\varPhi }{{dt}}=\frac{N\pi {a}^{3}}{{l}_{{ic}}}\eta \,\cdot\, \left(\frac{d{B}_{0z}\left(t\right)}{{dt}}\,\cdot\, D\left(t\right)+{B}_{0z}\left(t\right)\,\cdot\, \frac{{dD}\left(t\right)}{{dt}}\right)$$

where

8 $$\eta =\frac{\left(2{\mu }_{r}+1\right){ka}{J}_{\frac{3}{2}}\left({ka}\right)-{k}^{2}{a}^{2}{J}_{\frac{1}{2}}\left({ka}\right)}{\left({\mu }_{r}-1\right){ka}{J}_{\frac{3}{2}}\left({ka}\right)+{k}^{2}{a}^{2}{J}_{\frac{1}{2}}\left({ka}\right)},D=\left(\frac{{z}_{2}}{\sqrt{{R}_{{ic}}^{2}+{z}_{2}^{2}}}-\frac{{z}_{1}}{\sqrt{{R}_{{ic}}^{2}+{z}_{1}^{2}}}\right),{z}_{1}={l}_{s}-{\frac{1}{2}l}_{{ic}}-{vt},{z}_{2}={z}_{1}+{l}_{{ic}}$$

Suppose the coil excitation is a sinusoidal signal. In that case, Eq. (10) defines an amplitude-modulated signal, and the characteristic signal generated by the motion particle can be extracted through phase-sensitive detection. To quickly describe the signal processing process, the raw signal can be expressed as

9 $$S\left(t\right)=M\left(t\right)\sin \left(\omega t+{\varphi }_{1}\right)+N\left(t\right)$$

where $M(t)$ is the wear particle characteristic signal, $\omega$ is the excitation frequency, and $N\left(t\right)$ is the noise signal.

The reference signal with the same frequency can be expressed as

10 $${S}_{r}(t)=Q\sin \left(\omega t+{\varphi }_{2}\right)$$

By multiplying (12) and (13):

$$S\left(t\right)\times {S}_{r}\left(t\right)$$

11 $$\begin{array}{l}=\frac{1}{2}M\left(t\right)Q\cos \left({\varphi }_{1}-{\varphi }_{2}\right)-\frac{1}{2}M\left(t\right)Q\cos \left(2\omega +{\varphi }_{1}+{\varphi }_{2}\right)\\\,+\,N\left(t\right)Q\sin \left(\omega t+{\varphi }_{2}\right)\end{array}$$

High-frequency components can be filtered through low-pass filtering, and if ${\varphi }_{1}={\varphi }_{2}$, then only components related to particle characteristics remain in the output

12 $${S}_{o}=\frac{1}{2}M\left(t\right)Q$$

As shown in Fig. 1c, a spherical reflector is located at the origin of the spherical coordinate system $(r,\theta ,\varphi ),$ a planar ultrasound wave is incident along the z-axis, and the sound pressure ${P}_{i}$ and normal velocity ${v}_{{ir}}$ of the incident wave can be expressed as

13 $$\begin{array}{ll}{P}_{i}={p}_{0i}\left({\omega }_{0}\right)\exp \left({\rm{i}}{k}_{0}r\cos \theta \right)\\\qquad={p}_{0i}\left({\omega }_{0}\right)\mathop{\sum}\limits_{m=0}^{\infty }{{\rm{i}}}^{m}\left(2m+1\right){j}_{m}({k}_{0}r){{\rm{P}}}_{m}\left(\cos \theta \right)\end{array}$$

14 $${v}_{{ir}}=\frac{{p}_{0i}\left({\omega }_{0}\right)}{{\rm{i}}{\rho }_{0}{c}_{0}}\mathop{\sum }\limits_{m=0}^{\infty }{{\rm{i}}}^{m}\left(2m+1\right){{\rm{P}}}_{m}\left(\cos \theta \right)\frac{d{j}_{m}\left({k}_{0}r\right)}{d\left({k}_{0}r\right)}$$

where ${\omega }_{0}$ is the ultrasonic excitation frequency, ${\rho }_{0}$ is the medium density, ${c}_{0}$ is the sound speed in the medium, ${k}_{0}={\omega }_{0}/{c}_{0}$, ${j}_{l}\left(\cdot \right)$ is the spherical Bessel function, and ${{\rm{P}}}_{m}\left(\cdot \right)$ is the Legendre polynomial.

The scattered wave also has a polar-axis symmetric property, i.e., the amplitude of the sound pressure at any point on the sphere is independent of the direction angle $\varphi$. Therefore, the scattered wave sound pressure ${P}_{s}$ and normal velocity ${v}_{{sr}}$ can also be expressed as a superposition form of the spherical function:

15 $${P}_{s}={p}_{0i}\left({\omega }_{0}\right)\mathop{\sum }\limits_{m=0}^{\infty }{B}_{m}{h}_{m}^{\left(1\right)}\left({k}_{0}r\right){{\rm{P}}}_{m}\left(\cos \theta \right)$$

16 $${v}_{{sr}}=\frac{1}{{\rm{i}}{\rho }_{0}{c}_{0}}\frac{\partial {P}_{s}}{\partial \left({k}_{0}r\right)}=\frac{{p}_{0i}\left({\omega }_{0}\right)}{{\rm{i}}{\rho }_{0}{c}_{0}}\mathop{\sum}\limits_{m=0}^{\infty }{B}_{m}{{\rm{P}}}_{m}\left(\cos \theta \right)\frac{d{h}_{m}^{\left(1\right)}\left({k}_{0}r\right)}{d\left({k}_{0}r\right)}$$

where ${h}_{m}\left(\cdot \right)$ is the spherical Hankel function.

Solid particles in liquid can be viewed as rigid spheres fixed, and the boundary condition of a zero normal velocity on the sphere can be given as

17 $${\left[{v}_{{ir}}={v}_{{sr}}\right]}_{r=a}=0$$

In the context of bubbles within liquids, the boundary conditions entail the continuity of acoustic pressure, and the normal velocity on the sphere can be expressed as

18 $${\left.P\left(r,\theta ,\omega \right)\right|}_{r=a}={\left.{P}_{e}\left(r,\theta ,\omega \right)\right|}_{r=a}$$

19 $${\left.\frac{1}{{\rm{i}}{\rho }_{0}\omega }\frac{\partial P\left(r,\theta ,\omega \right)}{\partial r}\right|}_{r=a}={\left.\frac{1}{{\rm{i}}{\rho }_{e}\omega }\frac{\partial {P}_{e}\left(r,\theta ,\omega \right)}{\partial r}\right|}_{r=a}$$

By means of the boundary conditions, the sound pressure of the scattered wave of the particles in the liquid ${P}_{{sp}}$ and the sound pressure of the scattered wave of the bubbles in the liquid ${P}_{{sb}}$ can be obtained as follows:

20 $${P}_{{sp}}=-{p}_{0i}\left({\omega }_{0}\right)\mathop{\sum}\limits_{m=0}^{\infty }{{\rm{i}}}^{m}\left(2m+1\right)\frac{{j}_{m}^{{\prime} }\left({k}_{0}a\right)}{{h}_{m}^{{\prime} \left(1\right)}\left({k}_{0}a\right)}{h}_{m}^{\left(1\right)}\left({k}_{0}r\right){{\rm{P}}}_{m}\left(\cos \theta \right)$$

21 $${P}_{{sb}}=-{p}_{0i}\left({\omega }_{0}\right)\mathop{\sum }\limits_{m=0}^{\infty }\left(2m+1\right){{\rm{i}}}^{m}\frac{{j}_{m}^{{\prime} }\left({k}_{0}a\right)+{\rm{i}}{\beta }_{m}{j}_{m}\left({k}_{0}a\right)}{{h}_{m}^{{\prime} \left(1\right)}\left({k}_{0}a\right)+{\rm{i}}{\beta }_{m}{h}_{m}^{\left(1\right)}\left({k}_{0}a\right)}{h}_{m}^{\left(1\right)}\left({k}_{0}r\right){{\rm{P}}}_{m}\left(\cos \theta \right)$$

where ${\beta }_{m}={\rm{i}}{\gamma }_{e}\frac{{j}_{m}^{{\prime} }\left({k}_{e}a\right)}{{j}_{m}\left({k}_{e}a\right)},{\gamma }_{e}=\frac{{\rho }_{0}{c}_{0}}{{\rho }_{e}{c}_{e}},{k}_{e}={\omega }_{0}/{c}_{e}$, ${\rho }_{e}$ is the scatterer density, and ${c}_{e}$ is the speed of sound in the scatterer.

The Doppler effect states that a wave’s received frequency increases as the source moves toward an observer and decreases as the source moves away from an observer. The ultrasonic transducer emits a signal at a fixed frequency ${f}_{0}$. When encountering a contaminant, the resulting echo signal undergoes a frequency shift ${f}_{d}$ due to the Doppler effect, allowing for the expression of the relationship between the frequency shift and velocity:

22 $${f}_{d}=\frac{2{f}_{0}{vcos}\alpha }{{c}_{0}},v=\frac{{c}_{0}}{2{f}_{0}\cos \alpha }\,\cdot\, {f}_{d}$$

where $\alpha$ is the angle between the incident direction of the sound wave and the direction of the particle motion and *v* is the target velocity.

**Fig. 2:**
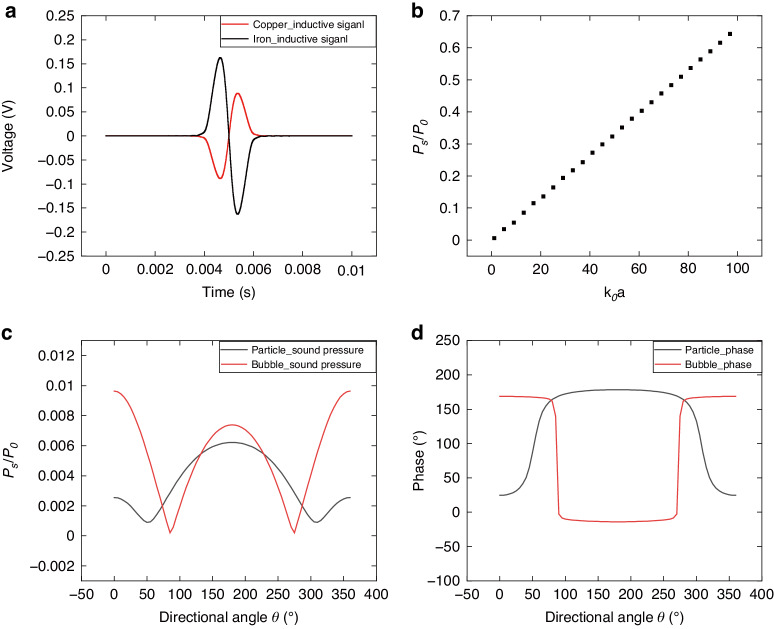
Verification of the detection principle through mathematical models. **a** Inductive signals for iron and copper particles in the reverse phase, **b** effect of the ultrasonic incident wavenumber and sphere size on the scattered waves, and **c**, **d** ultrasonic scattered waves at different directional angles for the particles and bubbles

In Fig. 2a, the induced voltages of the iron and copper particles are depicted in opposite phases. This phase difference facilitates the discrimination between the metal particles of distinct materials. Figure 2b illustrates that increased incident wave frequency and sphere size enhance the sound pressure of the scattered wave. A comprehensive analysis of Fig. 2c, d suggests that the optimal monitoring angle is 180°, i.e., reflection. This conclusion is drawn based on the relatively high scattered sound pressure observed at this angle, coupled with the fact that the waves emanating from the particle and the bubble are in opposite phases for easy differentiation.

### Finite element simulation

**Fig. 3:**
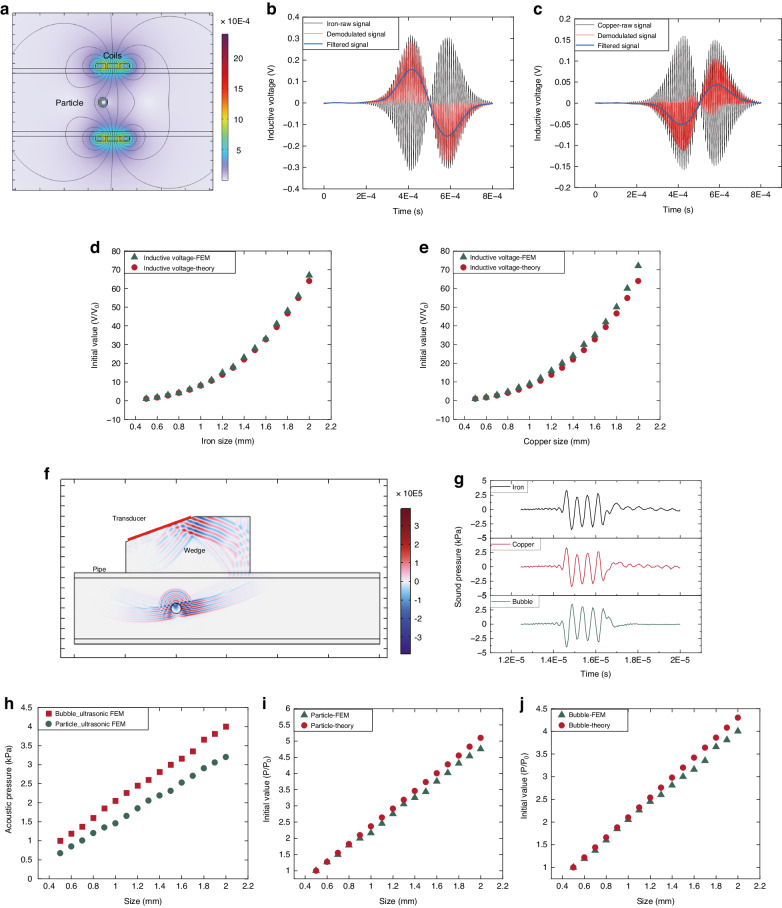
Inductive and ultrasonic simulation results. **a** Inductive simulation model, **b**, **c** copper and iron particle inductive simulation: raw and demodulated signals, **d**, **e** inductive signal amplitude under different particle sizes, and **f** ultrasonic simulation model, **g** iron, copper and bubble ultrasonic simulation results, and **h**–**j** ultrasonic signal amplitude under different sizes

In this chapter, electromagnetic induction and ultrasonic scattering simulations are executed separately. The feasibility of the proposed sensing method is verified through simulation, wherein the impact of various types and sizes of contaminants on induced and ultrasonic signals is systematically discussed. Figure 3 presents a simulation scenario involving spherical contaminants moving along the axis of the sensing unit, with a pipe diameter of 14 mm, a wall thickness of 1 mm, plexiglass material, and water as the medium.

In Fig. 3a, the induction simulation is depicted, employing coils with thicknesses of 2 mm and 100 turns. The excitation frequency is set at 100 kHz with an amplitude of 10 V, and both side coils are excited in an inverted phase. In Fig. 3b, c, the raw inductive signal for iron and copper particles manifests as an amplitude-modulated signal. After demodulation and filtering, a low-frequency signal that characterizes the particle information is extracted. The peak-to-peak value of the processed signal is used to estimate the particle size, while the phase provides information regarding the particle material. Figure 3d, e demonstrate that the induced voltage amplitude is approximately proportional to the cube of the particle size, aligning with the theoretical expectations discussed above.

Figure 3f illustrates the ultrasonic simulation, featuring an incident wave frequency of 2 MHz. In Fig. 3g, the raw ultrasonic signals from iron and copper particles exhibit nearly identical characteristics, while the signals from bubbles are in the opposite phase. Figure 3h–j further illustrate that the sound pressure is roughly proportional to the size of the contaminants, aligning with the theoretical framework discussed earlier.

### Experiments

**Fig. 4:**
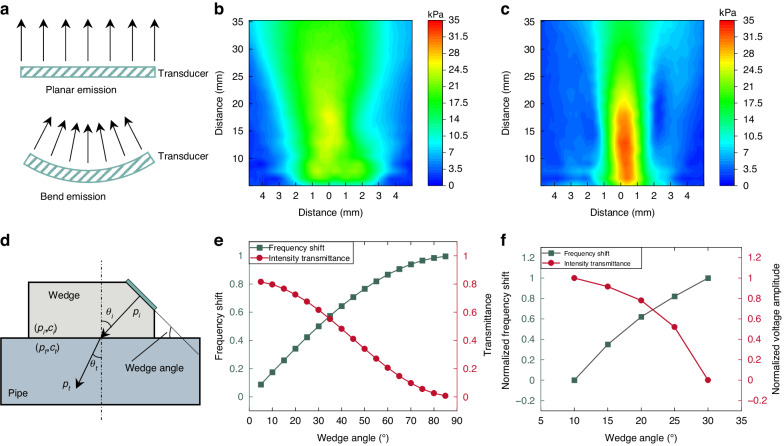
Flexible transducer sound field characteristics. **a** Sound field focusing through flexible bending, **b** sound field in water for planar emission, **c** sound field in water for 14 mm curvature radius bend emission, **d** sound transmission schematic, **e** theoretical optimization of the wedge angle, and **f** experimental optimization of the wedge angle

As shown in Fig. 4a–c, the acoustic field undergoes a focusing effect when the flexible ultrasonic patch is bent with a curvature radius of 14 mm. In this situation, the acoustic pressure is notably higher than its emission in a planar state. This focused acoustic field has proven to be advantageous for the enhanced detection of contaminants within the pipeline.

The incidence angle of sound waves significantly influences both the Doppler frequency shift and the transmittance of sound waves. The relationship between Doppler frequency shift and incidence angle has been elucidated earlier. The acoustic transmittance ${t}_{p}$ between two media can be expressed as

23 $$\begin{array}{c}{t}_{p}\equiv \frac{{p}_{t}\left(\omega \right)}{{p}_{i}\left(\omega \right)}=\frac{2m\cos {\theta }_{i}}{m\cos {\theta }_{i}+\sqrt{{n}^{2}-{\sin }^{2}{\theta }_{i}}},m=\frac{{\rho }_{t}}{{\rho }_{i}},n=\frac{{c}_{i}}{{c}_{t}}\end{array}$$

Figure 4d illustrates the process of sound wave propagation. Figure 4e demonstrates that an increase in the wedge angle corresponds to a decrease in transmittance, accompanied by an increase in the Doppler frequency shift generated by contaminants moving along the axis. Reduced transmittance implies diminished sound pressure penetrating the pipe, which is unfavorable for effectively detecting contaminants within the pipe. On the other hand, a more significant Doppler frequency shift facilitates the detection of contaminant movement speed. Consequently, selecting an appropriate wedge angle becomes crucial, considering both the transmittance and the frequency shift. Figure 4f shows the experimental results of the optimization process for the wedge angle. Based on the theoretical and experimental results, the wedge angle is 20 degrees.

**Fig. 5:**
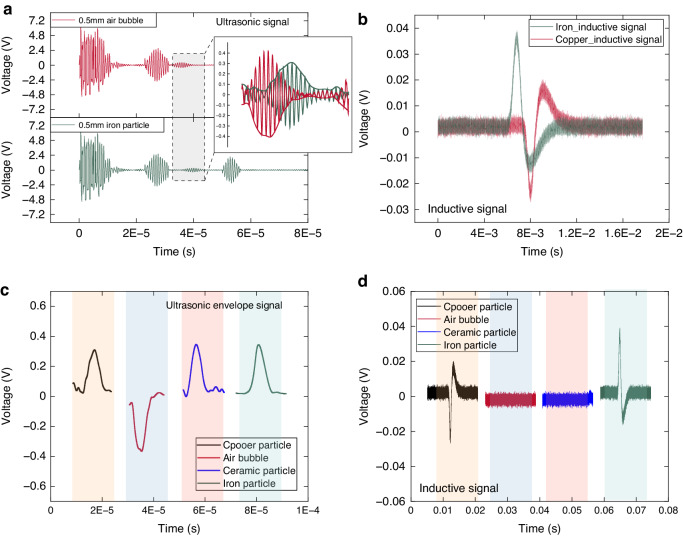
Experimental results for contaminant type differentiation. **a** ultrasonic signals of bubbles and particles in the reverse phase, **b** inductive signals for iron and copper particles in the reverse phase, **c** ultrasonic signals of different contaminants, and **d** inductive signals of different contaminants

Figure 5 presents the experimental results for various types of contaminants. As depicted in Fig. 5a, the ultrasonic signals from the particles and bubbles exhibit a reverse phase, and the characteristic reflector signals can be derived through envelope processing. Figure 5b displays the electromagnetic induction signal of the particles. Figure 5c, d illustrate the ultrasonic and electromagnetic induction signals of the copper particles, iron particles, ceramic particles, and bubbles. Their signal characteristics are consistent with those proposed above, underscoring the effectiveness of the proposed method in distinguishing between different types of contaminants.

**Fig. 6:**
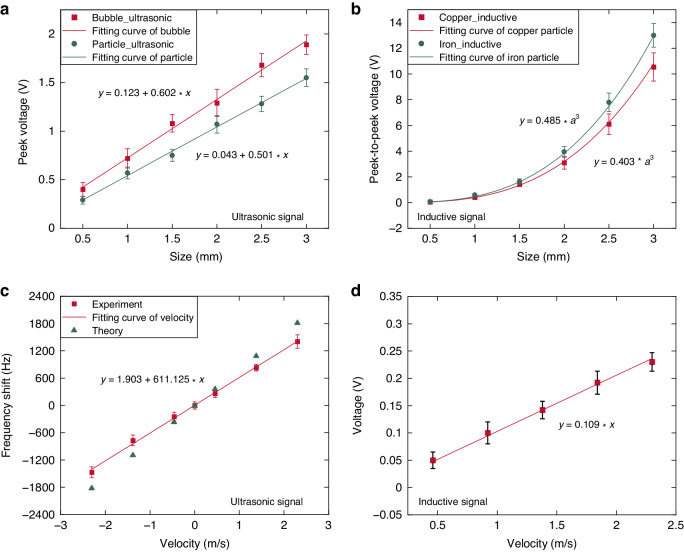
Experimental results for different contaminant sizes and velocities. **a** Ultrasonic signals at different sizes, **b** inductive signals at different sizes, **c** Doppler shift at different velocities, and **d** inductive signals at different velocities

Figure 6 illustrates the experimental results of ultrasonic and electromagnetic inductive sensing experiments at varying sizes and speeds. The experimental conditions in Fig. 6a, b include a constant flow rate (0.46 m/s) and varying particle and bubble sizes. Figure 6c, d show the effect of different flow rates on a 0.5 mm diameter copper particle. In Fig. 6a, the amplitude of the ultrasonic signals from the particles and bubbles is approximately proportional to the size of the contaminant. Additionally, Fig. 6b reveals that the inductive signal amplitude is proportional to the cube of the metal particle size. These observed relationships align with the theoretical analysis and simulations above. Figure 6c depicts the proportional relationship between the contaminant velocity and the Doppler shift, forming the basis for flow velocity sensing. Furthermore, Fig. 6d suggests that the inductive signal is roughly proportional to the particle velocity. Given that the speed of particle movement in a pipeline is often considered equivalent to the fluid flow rate, variations in the pipeline flow rate substantially influence the output of the induction signal, consequently impacting the detection accuracy. Therefore, velocity compensation is necessary.

Via the traditional method, the electromagnetic induction sensor is calibrated at a set flow rate, and only the fitted curve, as shown in Fig. 6b of the manuscript, is obtained without flow rate information in the voltage solution. However, systems do not maintain the same flow velocity at all times. Figure 6d shows that the voltage is proportional to the flow velocity. Therefore, if the flow speed changes, then adhering to the original calibration equation will lead to mistakes. This is where the ultrasonic Doppler effect occurs. This paper suggests using this effect to monitor flow speed in real time and then adding it to the calibration equation to fix those errors.

Taking this work as an example, the traditional inductive calibration equation based on Fig. 6b is $u=M\,\cdot\, {a}^{3}$, where $M=0.403$. In Fig. 6d, the induced voltage is proportional to the velocity ${u}_{v}=Q* v$, where $Q=0.109$. The flow velocity *v* is introduced for compensation; thus, the calibration equation is expressed as ${u}_{c}={Nv}\,\cdot\, {a}^{3}$. When the experimental conditions of Fig. 6b, d are simultaneously satisfied, ${\left.{u}_{v}={u}_{c}\right|}_{v=0.46,a=0.5}$. The calibration equation is finally obtained ${u}_{c}=0.872* v* {a}^{3}$. The proposed method utilizes the Doppler effect to measure flow velocity during contaminant detection, which provides the possibility for accuracy compensation.

## Materials and Methods

### Processing of the micromachined flexible ultrasonic transducer patch

**Fig. 7:**
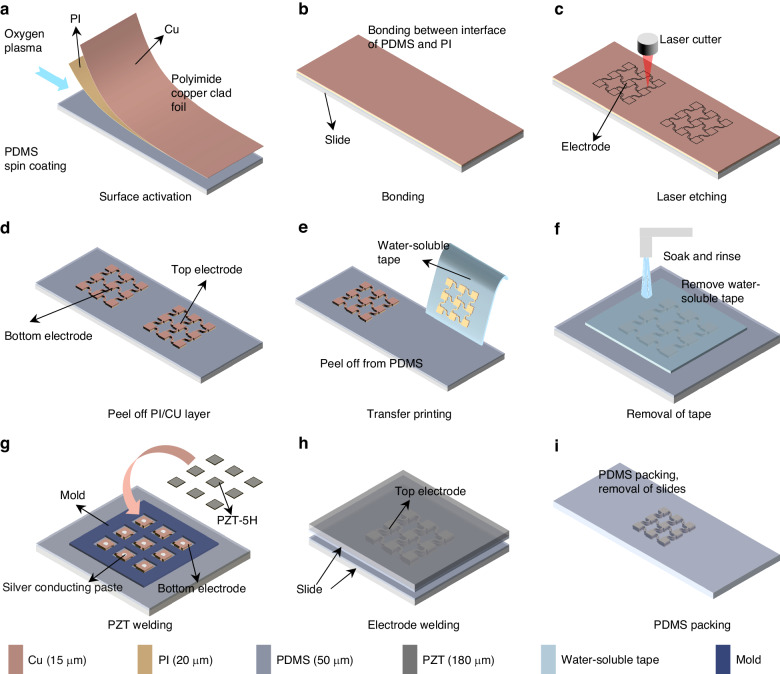
Flow chart for processing the micromachined flexible ultrasonic transducer patch. **a** Surface activation, **b** Bonding, **c** Laser etching, **d** Peel off PI/CU layer, **e** Transfer printing, **f** Removal of tape, **g** PZT welding, **h** Electrode welding, **i** PDMS packing

Figure 7 shows the processing flow of the micromachined ultrasonic flexible patch. A layer of 50 μm polydimethylsiloxane (PDMS) is initially spin-coated on the slide with a spin-coating speed of 1000 rpm, and then the PDMS is thermally cured at 80 °C for one hour. The surfaces of the PDMS and polyimide copper-clad foil are activated by oxygen plasma treatment and then bonded. Laser etching (1.65 W power, 25 kHz pulse repetition frequency, 400 mm/s laser cutting speed, 1000 ns pulse width, 15 μm etch line spacing, and 350 etching times) is employed to create the top and bottom electrodes. The electrodes and PZT-5H are laser cut into 6*6 arrays with an array element size of 700 μm and a serpentine electrode width of 150 μm between the array elements. Then, the PI (20 μm) and CU (15 μm) layers are subsequently removed. Water-soluble tape (the main component of which is polyvinyl alcohol (PVA)) is then used to peel and transfer the electrodes to another spin-coated PDMS slide. A mold is used to assist in aligning the PZT-5H (180 μm) and bottom electrode, and then they are bonded by heating and curing the silver conductive paste. The top electrode and PZT-5H are aligned by hand under an optical microscope and then are bonded with silver conductive paste. After PDMS packaging to fill gaps and then slide removal, a micromachined flexible ultrasonic transducer patch is processed.

### Experimental procedures

**Fig. 8:**
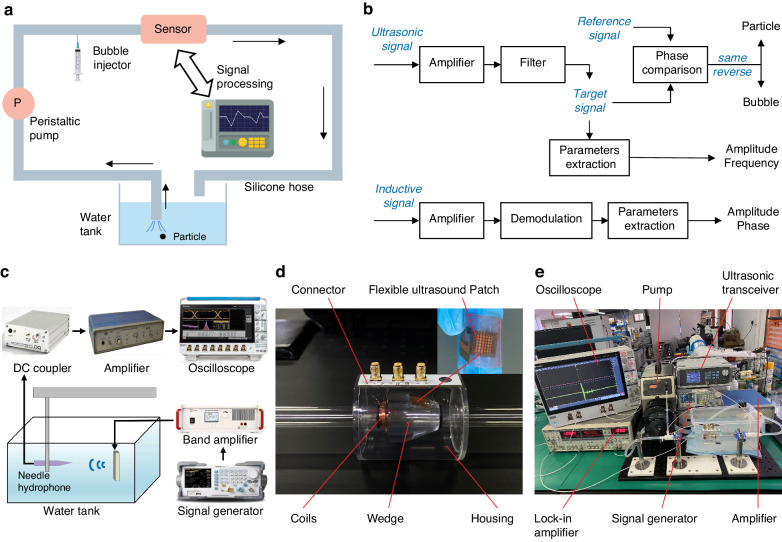
Experimental procedure and equipment. **a** Schematic of the experimental procedure, **b** signal processing of the ultrasonic and inductive units, **c** acoustic field measurement schematic, **d** sensor prototype, and **e** experimental bench

Figure 8a shows a schematic of the experimental procedure. A particle is introduced into a water tank and drawn into the pipe using a peristaltic pump. It is passed through the sensor, discharged from the pipe outlet back to the tank, and so on for the experiment. Moreover, a syringe gradually introduces air into the pipe to generate bubbles. Following the passage of the contaminant through the sensor, the raw signal is captured and processed by the relevant experimental instruments.

Figure 8b illustrates the signal processing procedure. The raw ultrasound signal undergoes amplification and filtering to isolate the desired signal. By comparing the phase of the target signal with that of the reference signal (i.e., the precollected particle signal), the nature of the target (particle or air bubble) is determined. This information is then utilized to establish the upper and lower envelopes. Additionally, spectral analysis is employed to extract the amplitude and frequency of the target signal, enabling the determination of the target size and flow rate. Simultaneously, the inductive raw signal undergoes demodulation via a lock-in amplifier after preamplification. The signal amplitude and phase are further analyzed spectrally to determine the target size and material. Ultimately, the outcomes obtained from the ultrasound and induction units are combined to identify the target material based on the type of judgment rule depicted in Fig. 1f.

Figure 8c shows a schematic of the acoustic field measurement. To obtain the results of the acoustic performance experiments. A broadband needle hydrophone is used to measure the sound field of the ultrasonic patch in water. Ultrasonic excitation is generated by a signal generator and band amplifier. A hydrophone is used to receive the ultrasound signals, which are captured by an oscilloscope through a DC coupler and an amplifier.

Figure 8d, e represent the sensor prototype and the experimental bench, respectively. The sensor consists of an inductive unit and an ultrasonic unit. The inductive unit contains three symmetrically arranged hollow coils. The two side coils are excited inversely, and the intermediate coil generates an inductive signal. The ultrasonic unit is a flexible patch with flexibility and bendability. The ultrasonic patch is attached to a wedge by a coupling agent, and the coupling agent is also applied between the wedge and the pipe.

### Material and parameter settings in the simulations and experiments

The simulations in this work are conducted via the COMSOL simulation platform. Inductive sensing simulations involve the consideration of physical fields such as AC/DC electromagnetic fields and moving meshes. Alternatively, ultrasonic sensing simulations are based on physical fields, including acoustics, pressure acoustic transients, and dynamic meshes. Specifically, the coil material is copper, while the pipe and wedge are made of plexiglass. Additionally, water is utilized as the medium within the pipe.

In the flexible ultrasonic patch process, PDMS (DC184; Dow Chemistry), a piezoelectric disk (PZT-5H; Changzhou Ultrasonic Electronics Co., Ltd.), polyimide copper-clad foil (Advanced Institute Technology Co., Ltd., Shenzhen), and water-soluble tape (is from Tesa) are utilized. A FemtoYL-40 laser ablation system, a WS-650Mz-23NPPB spin coater, and a DZH-6050 model drying vacuum oven are also used in this process.

For the acoustic field measurements, a needle hydrophone (PA-NH2000), a DC coupler (KEITHLEY-2612A), a signal generator (RIGOL-DG1032), an amplifier (RITEC-BR640), and an oscilloscope (Tektronix-MSO064) are employed.

In the experiments, a 1 mm thick pipe with a 14 mm outer diameter is utilized. The coils, which consisted of an enameled copper wire with a 14 mm diameter and 100 turns, are wound around the pipe using a BAS-01-30 winding machine. The ultrasonic patch is a 6*6 array structure with a 10*10 mm size. A BR8000Y353 peristaltic pump is used for drawing particles into the pipe. The inductive excitation is a 10 V, 100 kHz sine wave; the signal generator utilized is model RIGOL-DG1032. An RITEC-BR640 amplifier and an SRS-SR844 lock-in amplifier are also employed. The ultrasonic excitation is a −100 V, 2 MHz pulsed wave, with a 500 ns pulse width and a 100 Hz pulse repetition frequency. A CTS-8077PR ultrasonic signal transceiver is used for these measurements.

## Conclusion

We successfully demonstrated an integrated ultrasonic-inductive sensor for the multiparameter detection of pipe contaminants. The flexible ultrasonic transducer patch, fabricated through micromachining technology, offers enhanced ease of installation on pipelines and enables sound field focusing to improve detection performance. The electromagnetic induction unit, comprising three symmetrical coils generating a symmetrical anti-phase excited magnetic field, proves advantageous for detecting metal particles. Mathematical models for ultrasonic scattering sound pressure and electromagnetic induction voltage were separately established, theoretically substantiating the feasibility of the proposed detection principle. Finite element simulations for ultrasonic and inductive sensing were conducted, validating the correctness of the theoretical model. Optimization of the ultrasonic incident angle was achieved through experiments and theoretical derivation. By employing ultrasonic excitation at 2 MHz and electromagnetic excitation at 100 kHz, diverse contaminants were tested in a 14 mm diameter pipe. The experimental results indicate that through a comprehensive analysis of ultrasonic and induction signals, the proposed method can be used to effectively distinguish various types of contaminants, including magnetic metal particles (e.g., iron), nonmagnetic metal particles (e.g., copper), nonmetallic particles (e.g., ceramics), and bubbles. This capability can cover almost all conceivable types of contaminants present in pipeline systems. Furthermore, this sensor demonstrated the ability to judge the size of contaminants based on signal amplitude, successfully detecting spherical contaminants ranging from 0.5 to 3 mm. Additionally, accurate flow velocity measurements through ultrasonic Doppler frequency shifts are emphasized, contributing significantly to detection accuracy compensation and equipment status monitoring. In conclusion, this research introduces a real-time online contaminant multiparameter monitoring method to measure almost all contaminant types and flow rates in pipelines. The implications of this study can be extended to diverse fields, such as industrial, aerospace, and clinical medicine, underscoring its broader significance.

## References

[1] Li, Z., Meng, Z. & Gibson, A. Detection of Nonmetallic Contaminants in Lubricating Oil Using a Microwave Rectangular Cavity Resonator Sensor. *IEEE Trans. Instrum. Meas.* **72**, 1–10, 10.1109/tim.2023.3293876 (2023).37323850 10.1109/tim.2023.3293876

[2] Yang, S., Cao, N. & Yu, B. Wear debris measurement in lubricating oil based on inductive method: A review. *Meas. Control* **56**, 1422–1435, 10.1177/00202940231159117 (2023). 10.1177/00202940231159117

[3] Sun, J. et al. Online oil debris monitoring of rotating machinery: A detailed review of more than three decades. *Mech. Syst. Sig. Processing* **149**, 10.1016/j.ymssp.2020.107341. (2021).

[4] Jia, R., Wang, L., Zheng, C. & Chen, T. Online Wear Particle Detection Sensors for Wear Monitoring of Mechanical Equipment—A Review. *IEEE Sens. J.* **22**, 2930–2947, 10.1109/jsen.2021.3131467 (2022). 10.1109/jsen.2021.3131467

[5] Wakiru, J. M., Pintelon, L., Muchiri, P. N. & Chemweno, P. K. A review on lubricant condition monitoring information analysis for maintenance decision support. *Mech. Syst. Signal Process.* **118**, 108–132, 10.1016/j.ymssp.2018.08.039 (2019). 10.1016/j.ymssp.2018.08.039

[6] Zhu, X., Zhong, C. & Zhe, J. Lubricating oil conditioning sensors for online machine health monitoring – A review. *Tribology Int.* **109**, 473–484, 10.1016/j.triboint.2017.01.015 (2017). 10.1016/j.triboint.2017.01.015

[7] Hong, W., Cai, W., Wang, S. & Tomovic, M. M. Mechanical wear debris feature, detection, and diagnosis: A review. *Chin. J. Aeronautics* **31**, 867–882, 10.1016/j.cja.2017.11.016 (2018). 10.1016/j.cja.2017.11.016

[8] Yang, D. & Liu, X. State-of-Art of Metal Debris Detection in Online Oil Monitoring. in Proceedings of IncoME-VI and TEPEN 2021, (Mechanisms and Machine Science, 2023, ch. Chapter 26, 307–314.

[9] Liu, Z., Zuo, H., Liu, Y., Li, X. & Chen, Z. Research on Wear Particle Online Monitoring Using Machine Vision for Rotating Machinery. *IEEE Trans. Instrum. Meas.* **72**, 1–11, 10.1109/tim.2023.3282677 (2023).37323850 10.1109/tim.2023.3282677

[10] Li, B., Xi, Y. H., Feng, S., Mao, J. H. & Xie, Y. B. A direct reflection OLVF debris detector based on dark-field imaging. *Measurement Sci. Technol.* **29**, 065104, 10.1088/1361-6501/aab9fc (2018).

[11] Li, B. et al. A Full Field-of-View Online Visual Ferrograph Debris Detector Based on Reflected Light Microscopic Imaging. *IEEE Sens. J.* **21**, 16584–16597, 10.1109/jsen.2021.3079174 (2021). 10.1109/jsen.2021.3079174

[12] Feng, S. et al. Wear Debris Segmentation of Reflection Ferrograms Using Lightweight Residual U-Net. *Ieee Transac. Instrumentation Measurement.* **70**, Art no. 5013611, 10.1109/tim.2021.3099573. (2021).

[13] Shi, H. et al. Comprehensive detection method for multi-contaminants in hydraulic oil based on inductance-resistance-capacitance analysis. *Tribol. Int.* **173**, 10.1016/j.triboint.2022.107609. (2022).

[14] Han, Z., Wang, Y., & Qing, X. Characteristics Study of In-Situ Capacitive Sensor for Monitoring Lubrication Oil Debris. *Sensors*. **17**, 10.3390/s17122851. (2017). 10.3390/s17122851 PMC5751668 29292748

[15] Xu, C., Zhang, P., Wang, H., Li, Y. & Lv, C. Ultrasonic echo waveshape features extraction based on QPSO-matching pursuit for online wear debris discrimination. *Mech. Syst. Signal Process.* **60-61**, 301–315, 10.1016/j.ymssp.2015.01.002 (2015). 10.1016/j.ymssp.2015.01.002

[16] Xu, C. et al. Discriminating debris particle in lubricant by ultrasonic waveshape features. *Ind. Lubrication Tribology* **67**, 202–209, 10.1108/ilt-03-2013-0033 (2015). 10.1108/ilt-03-2013-0033

[17] Feng, S. et al. Sensing Model for Detecting Ferromagnetic Debris Based on a High-Gradient Magnetostatic Field,” Ieee-Asme. *Trans. Mechatron.* **27**, 2440–2449, 10.1109/tmech.2021.3114002 (2022). 10.1109/tmech.2021.3114002

[18] Zhang, W. et al. Motion simulation analysis of wear debris in an integrated detection unit for lubricating oil. *Eng. Appl. Comput. Fluid Mech.* **17**, Art no. 2255035, 10.1080/19942060.2023.2255035. (2023).

[19] Novak, N., Trajkovski, A., Polajnar, M., Kalin, M., & Majdič, F. Wear of hydraulic pump with real particles and medium test dust. *Wear*. 532–533, 10.1016/j.wear.2023.205101. (2023).

[20] Ilerioluwa, L. et al. A Multi-Parameter Microfluidic Particle Sensor Based on Permalloy for High Sensitivity. *IEEE Trans. Instrum. Meas.* **71**, 1–10, 10.1109/tim.2022.3154795 (2022). 10.1109/tim.2022.3154795

[21] Shi L., Qu, M., Lv, D., Liu, W., & Xie, J. A two-channel ultrasonic flowmeter based on AlN piezoelectric micromachined ultrasonic transducers arrays with improved cross-correlation method. *J. Micromech. Microeng.* **33**, 10.1088/1361-6439/ad0307. (2023).

[22] Zhang, H., Ma, L., Shi, H., Xie, Y. & Wang, C. A Method for Estimating the Composition and Size of Wear Debris in Lubricating Oil Based on the Joint Observation of Inductance and Resistance Signals: Theoretical Modeling and Experimental Verification. *IEEE Trans. Instrum. Meas.* **71**, 1–9, 10.1109/tim.2022.3179490 (2022). 10.1109/tim.2022.3179490

[23] Qian, M., Ren, Y. & Feng, Z. Wear Debris Sensor Using Intermittent Excitation for High Sensitivity, Wide Detectable Size Range, and Low Heat Generation. *IEEE Trans. Ind. Electron.* **70**, 6386–6394, 10.1109/tie.2022.3190894 (2023). 10.1109/tie.2022.3190894

[24] Shi, H. et al. Inductive-Capacitive Coulter Counting: Detection and Differentiation of Multi-Contaminants in Hydraulic Oil Using a Microfluidic Sensor. *IEEE Sens. J.* **21**, 2067–2076, 10.1109/jsen.2020.3016000 (2021). 10.1109/jsen.2020.3016000

[25] Shi, H. et al. An Integrated Inductive-Capacitive Microfluidic Sensor for Detection of Wear Debris in Hydraulic Oil. *IEEE Sens. J.* **19**, 11583–11590, 10.1109/jsen.2019.2936328 (2019). 10.1109/jsen.2019.2936328

[26] Wang, C. et al. An Oil Multipollutant Detection Sensor With High Sensitivity and High Throughput. *IEEE Trans. Instrum. Meas.* **71**, 1–11, 10.1109/tim.2022.3181293 (2022). 10.1109/tim.2022.3181293

[27] Xu, C. et al. Experimental Research on Detecting Ability of Novel Online Ultrasonic Wear Debris Sensor. *Lubrication Eng.* **41**, 21–25 (2016). Art no. 0254-0150(2016)41:1<21:Xxcsml>2.0.Tx;2-#. [Online]. Available: <Go to ISI>://CSCD:5622680.

[28] Du, L., & Zhe, J. An integrated ultrasonic–inductive pulse sensor for wear debris detection. *Smart Mater. Struc.* **22**, 10.1088/0964-1726/22/2/025003.(2013).

[29] Wu, X. et al. A New Inductive Debris Sensor Based on Dual-Excitation Coils and Dual-Sensing Coils for Online Debris Monitoring. *Sensors* **21**, 7556, 10.3390/s21227556 (2021). 34833634 10.3390/s21227556 PMC8624713

[30] Yuan, Z. et al. A Ferromagnetic Particle Sensor Based on a Honeycomb Permanent Magnet for High Precision and High Throughput. *IEEE Transac. Instrumentation Measurement*. 1–1, 2022, 10.1109/TIM.2022.3216401.

